# 0633. Time-resolved spectroscopy using non-invasive monitoring may detect hapatic ischemia

**DOI:** 10.1186/2197-425X-2-S1-P43

**Published:** 2014-09-26

**Authors:** Y Tomotsugu, Y Kakihana, K Yamaguchi, M Nakahara, T Futatsuki, J Taniguchi, K Nakamura, N Okayama

**Affiliations:** Kagoshima University Hospital, Intensive Care Medicine, Kagoshima University, Japan

## Introduction

The aim of the present study was to investigate whether the changes in hepatic oxygenation can be detected by time-resolved spectroscopy (TRS) placed on the skin surface above the liver.

## Methods

With approval of the local Hospital Ethics Committee and informed consent, 6 healthy volunteers aged 28.8 years (25-36 years), and 5 patients with chronic renal failure aged 70.6 years (58-81 years) were studied. In 6 healthy volunteers, following the echography, TRS (TRS-10, Hamamatsu Photonics K.K., Hamamatsu, Japan) probe consisting of a near-infrared light (at 760 nm, 800 nm, 835 nm) emitter and a receiver optode, was placed 4 cm apart on the abdominal skin surface above the liver or apart from the liver at least 10 cm. In 5 patients with chronic renal failure, following the echography, TRS probes were placed 4 cm apart on the skin surface above the liver during hemodialysis (HD).

## Results

In 6 healthy volunteers, the values of abdominal total hemoglobin concentration (tHb) was significantly higher in the liver area than in the other area (80.6±26.81mM vs 44.6±23.1mM, P=0.0017), while the value of abdominal SO_2_ in the liver area was nearly the same as that in the other area (71.5±3.6% vs 73.6±4.6%, P=0.19). The values of mean optical path length and scattering coefficient (µ´s) at 800 nm in the liver area were significantly different from those in the other area (21.3±4.9 cm vs 29.2±5 cm, p=0.0004, and 7.97±1.14cm-1vs 9.02±0.51cm-1, P=0.015). One of 5 patients with chronic renal failure complained of the severe abdominal pain during HD, and the abdominal SO_2_ was decreasing from 53% to 22%, but the pain relief occurred following cessation of HD, and SO_2_ recovered to the baseline.

## Conclusions

Our data suggest that the optical properties of the liver may be measured by the TRS placed on the skin surface, and the hepatic oxygenation may act as a non-invasive monitoring for early detection of intestinal ischemia.

